# MGRN: toward robust drug recommendation via multi-view gating retrieval network

**DOI:** 10.1093/bioinformatics/btae572

**Published:** 2024-09-24

**Authors:** Fanjun Meng, Xiaobo Li, Xiaodi Hou, Mingyu Lu, Yijia Zhang

**Affiliations:** School of Artificial Intelligence, Dalian Maritime University, 116026 Dalian, China; School of Information Science and Technology, Dalian Maritime University, 116026 Dalian, China; School of Artificial Intelligence, Dalian Maritime University, 116026 Dalian, China; School of Artificial Intelligence, Dalian Maritime University, 116026 Dalian, China; School of Information Science and Technology, Dalian Maritime University, 116026 Dalian, China

## Abstract

**Motivation:**

Drug recommendation aims to allocate safe and effective drug combinations based on the patient’s health status from electronic health records, which is crucial to assist clinical physicians in making decisions. However, the existing drug recommendation works face two key challenges: (i) difficulty in fully representing the patient’s health status leads to biased drug representation; (ii) only focusing on diagnostic representations of multiple visits, neglecting the modeling of patient drug history.

**Results:**

To address the above limitations, we propose a multi-view gating retrieval network (MGRN) for robust drug recommendation. We design visit-, sequence-, and token-level views to provide different perspectives on the interaction between patients and drugs, obtaining a more comprehensive representation of drugs. Moreover, we develop a gating drug retrieval module to capture critical drug information from multiple visits, which can assist in recommending more reasonable drug combinations for the current visit. When evaluated on publicly real-world MIMIC-III and MIMIC-IV datasets, the proposed MGRN establishes a new benchmark performance, particularly achieving improvements of 1.36%, 1.71%, 1.21% and 2.12%, 2.36%, 1.81% in Jaccard, PRAUC, and *F*1-score, respectively, compared to state-of-the-art models.

**Availability and implementation:**

The code is available at: https://github.com/kyosen258/MGRN.git.

## 1 Introduction

In recent years, due to the outbreak of large-scale infectious diseases and the scarcity and uneven distribution of medical resources, more and more patients are experiencing deterioration of their condition (Li *et al*. 2024). Therefore, clinical support systems based on artificial intelligence, such as drug recommendations (Sun *et al*. 2022), radiology report generation (Hou *et al*. 2023), and International Classification of Disease (ICD） coding (Li *et al*. 2023b), have received widespread attention and rapid development.

Drug recommendation involves modeling the feature representation of medical entity sequences (diagnosis and procedure) during visits in low-dimensional space and mining the intrinsic relationship between medical entity and drug to recommend accurate prescriptions (Chen *et al*. 2023). Previous methods mainly include instance-based methods (Zhang *et al*. 2017, Wang *et al*. 2018) and longitudinal-based methods (Wang *et al*. 2021a, 2021b, Ren *et al*. 2022). The instance-based methods focus more on the patient’s current health status and need to explore past medical information fully (Li *et al*. 2023c). For example, Zhang *et al*. (2017) employed a recurrent encoder to capture the dependency relationships between labels during the current visit and used a content-based attention mechanism to extract disease-drug mappings. Wang *et al*. (2018) used linear methods to integrate demographic, procedure, and diagnosis information for drug recommendation. However, the longitudinal-based methods utilize the time dependence of visit records to capture potentially helpful information between multiple visits to represent patients more comprehensively. Choi *et al*. (2016) used an attention-generation mechanism to capture sequential medical information. Shang *et al*. (2019) viewed longitudinal patient records as queries and combined drug and drug–drug interaction (DDI) graphs for memory enhancement.

**Figure 1.**
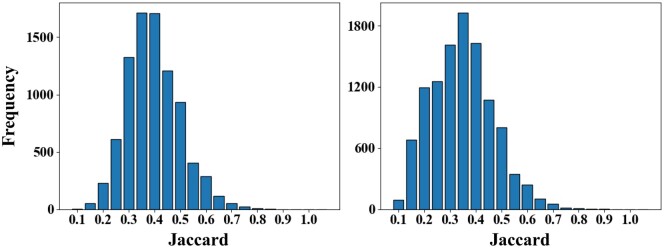
The histogram of Jaccard between current and history visit for each patient on the MIMIC-III (left) and MIMIC-IV (right), where Jaccard is a statistical indicator used to compare the similarity between two sample sets.

Although recent drug recommendation efforts have achieved competitive performance, some key issues must be addressed. On the one hand, previous models typically represent medical entity sequences and drug combinations separately and then perform later fusion, which will lose some critical features and information interactions (Wu *et al*. 2023). Meanwhile, modeling diagnostic and procedural representations solely from the sequence-level input perspective will result in coarse-grained and incomplete patient representation, affecting patient–drug interaction and recommendation performance. Moreover, previous models often obtain a global representation of patients by calculating the similarity of patient representations from multiple visits, which may be inaccurate and contain a lot of noise, leading to incorrect drug recommendations (Li *et al*. 2023a). On the other hand, in clinical practice, diverse diseases have a complex and long-lasting course, some patients may even be taking the same drugs for their entire lives. The perspective of predicting drug combinations solely based on patient representation is singular, ignoring the direct intrinsic relationship between the patient’s historical drugs and current drugs. Figure 1 shows the Jaccard distribution histograms of current and historical drugs in two datasets. The statistical results indicate that the patient’s medication history is similar to the drug currently sought in varying degrees.

Considering the above issues, we propose a multi-view gating retrieval network (MGRN) for robust drug recommendation. MGRN consists of a multi-view patient–drug interaction (MPI) module and a gating drug retrieval module. The former is responsible for patient–drug interaction from diverse perspectives to obtain a comprehensive and fine-grained patient representation and interactive feature of drugs. The latter aims to select valuable drugs from the patient’s historical drug set for the current visit. Overall, we conduct a series of experiments on two real-world benchmark datasets, i.e. MIMIC-III and MIMIC-IV, to assess the effectiveness of our model. The experimental results exceed the most competitive models on both datasets, achieving state-of-the-art (SOTA) performance. The ablation experiment, robustness experiment, and case analysis further validate the efficacy of our model.

The proposed contributions of MGRN are as follows:

- We design visit-, sequence-, and token-level views to provide different perspectives on the interaction between patients and drugs, obtaining a more comprehensive and fine-grained representation of drugs.
- We develop a gating drug retrieval module to capture critical drug information from multiple visits, which can assist in recommending more effective drug combinations for the current visit.
- We conduct extensive experiments on real-world MIMIC-III and MIMIC-IV datasets, and the results show that our proposed MGRN model achieves SOTA performance.

## 2 Materials and methods

### 2.1 Problem formulation

The electronic health record (EHR) of a patient $P={\left\{V^{t}\right\}}_{t=1}^{T}$ consists several visits $V^{t}=\{D^{t},P^{t},M^{t}\}$, where $D^{t}\in {\left\{0,1\right\}}^{N_{d}}$ is the diagnosis set, $P^{t}\in {\left\{0,1\right\}}^{N_{p}}$ is the procedure set and $M^{t}\in {\left\{0,1\right\}}^{N_{m}}$ is the drug set in the visit, $N_{d}$, $N_{p}$ and $N_{m}$ are the number of diagnoses, procedures, and drugs, respectively. *T* and *t* represent the total number of visits and the *t*-th visit of the patient, respectively. For each visit $V^{t}$, we need to utilize the patient’s current state $D^{t}$, $P^{t}$, along with their past medical records ${\left\{D^{i},P^{i}\right\}}_{i=1}^{t-1}$ and drug history ${\left\{M^{i}\right\}}_{i=1}^{t-1}$, to recommend appropriate drug combinations ${\hat{M}}^{t}$. In brief, we list the key notations in Supplementary Table S1.

### 2.2 Methods

**Figure 2.**
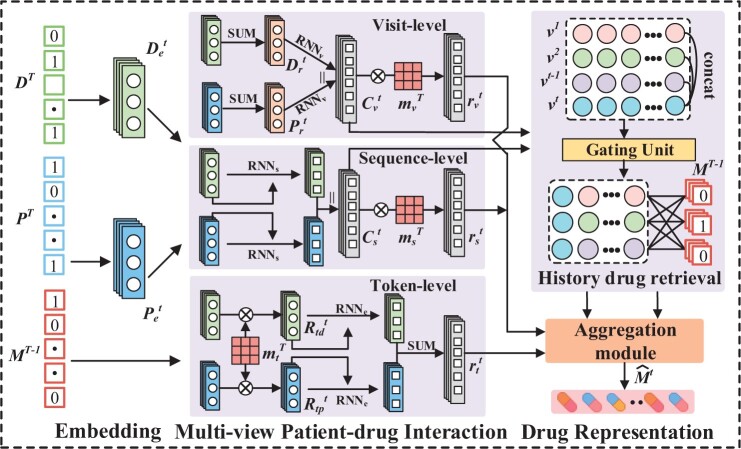
An overview of the present MGRN model. $D^{T}$, $P^{T}$, and $M^{T}$ denote the patient’s diagnose, procedure, and drug sets. $m^{T}$ denotes a randomly initialized drug representation. ⊗ and ‖ denote matrix multiplication and concat operation, respectively.

As shown in Fig. 2, MGRN consists of three components: embedding layer, MPI module, and drug representation (DR) module. The embedding layer projects the tokens in the diagnosis and procedure sets ${\left\{D^{t}\right\}}_{t=1}^{T}$ and ${\left\{P^{t}\right\}}_{t=1}^{T}$ into the embedding space. The MPI designs visit-, sequence-, and token-level views to provide different perspectives on the interaction between patients and drugs, obtaining a more comprehensive DR. The DR designs a gating drug retrieval strategy to choose the reusable drugs that are meaningful for the current visit, and then obtains the final drug combination by a aggregation module.

#### 2.2.1 Embedding layer

We take the diagnosis set ${\left\{D^{t}\right\}}_{t=1}^{T}$ and procedure set ${\left\{P^{t}\right\}}_{t=1}^{T}$ of each patient as input to the model, each set contains different combinations of medical entities, and then use the embedding method to map each set to a vector space. The formula is as follows:

(1) $$\begin{array}{c} \{\begin{array}{c} D_{e}^{t}={\text{Embedding}}_{d}(D^{t}),\\ P_{e}^{t}={\text{Embedding}}_{p}(P^{t}), \end{array} \end{array}$$

where $D_{e}^{t}=(d_{e}^{1},\ldots ,d_{e}^{sd})\in R^{sd\times d}$ and $P_{e}^{t}=(p_{e}^{1},\ldots ,p_{e}^{sp})\in R^{sp\times d}$, *sd* and *sp* represent the sequence lengths of diagnosis and procedure, respectively, while *d* represents the embedding dimension.

#### 2.2.2 Multi-view patient–drug interaction

Previous models learn diagnostic and procedural representations solely from the sequence-level input perspective, which results in coarse-grained and incomplete patient representation, affecting the recommendation of drug combinations. Moreover, many models typically represent medical entity sequences and drug combinations separately and then perform later fusion, which will lose some critical features and information interactions. Thus, we design visit-, sequence-, and token-level views to provide different perspectives on the interaction between patients and drugs, obtaining a more comprehensive DR.

**Visit-level view:** For certain long-term chronic diseases, there may be a significant correlation between patient longitudinal visits. Historical visit information can supplement critical feature for the current visit to obtain sufficient patient representation. Next, we interact the patient representation containing historical information with the DR to calculate the drug-matching score from a visit-level perspective.

Firstly, we sum the embeddings in the diagnosis $D_{r}^{t}\in R^{d}$ and procedure $P_{r}^{t}\in R^{d}$ sets to get the feature representation of each visit:

(2) $$\begin{array}{c} \{\begin{array}{c} D_{r}^{t}=\sum_{i=1}^{sd}d_{i}^{t},\\ P_{r}^{t}=\sum_{i=1}^{sp}p_{i}^{t}, \end{array} \end{array}$$

where *i* represents the embedding of the *i*-th medical entity.

Then, we utilize recurrent neural network (RNN) to encode the diagnosis and procedure of each visit and concat the encoded representations as the patient’s visit-level view:

(3) $$\begin{array}{c} \{\begin{array}{c} (D_{v}^{1},\ldots D_{v}^{T})={\text{RNN}}_{vd}((D_{r}^{1},\ldots D_{r}^{T})),\\ (P_{v}^{1},\ldots P_{v}^{T})={\text{RNN}}_{vp}((P_{r}^{1},\ldots P_{r}^{T})), \end{array} \end{array}$$

(4) $$\begin{array}{c} C_{v}^{t}=(D_{v}^{t}||P_{v}^{t}), \end{array}$$

where ‖ denotes contact operation, and $C_{v}^{t}$ is patient representation from the visit-level view.

Subsequently, we generate the drug-matching score representation for the visit-level perspective, as follows:

(5) $$r_{v}^{t}=C_{v}^{t}m_{v}^{T}$$

**Sequence-level view:** Multiple medical entities are involved in each diagnosis and procedure input sequence. These medical entities are interrelated and form the patient representation together. Therefore, capturing the long-term dependencies of medical entities in the input sequence is beneficial for obtaining a global representation of patients, providing a prerequisite for adequate interaction with DRs.

Based on the above considerations, we utilize bidirectional RNN to aggregate information from the diagnosis and procedure sets, which can model the long-term dependency relationship of input sequences and obtain a good patient representation, the process is as follows:

(6) $$\begin{array}{c} \{\begin{array}{c} D_{s}^{t}={\text{RNN}}_{sd}(d_{1}^{t},\ldots ,d_{sd}^{t}),\\ P_{s}^{t}={\text{RNN}}_{sp}(p_{1}^{t},\ldots ,p_{sp}^{t}), \end{array} \end{array}$$

(7) $$\begin{array}{c} C_{s}^{t}=(D_{s}^{t}||P_{s}^{t}), \end{array}$$

where $C_{s}^{t}$ denotes the patient representation for the sequence view.

Then, we obtain the matching drug score vectors from the sequence-level view, as follows:

(8) $$r_{s}^{t}=C_{s}^{t}m_{s}^{T}.$$

**Token-level view:** Compared to other perspectives, token-level views interact early between each medical entity in the input sequence and the DR, aiming to reduce information loss regarding entity–drug interaction during deep network propagation iterations and obtain entity-specific fine-grained DRs.

Therefore, we directly match each individual diagnose and procedure token vector with the representation vector of drugs $m_{t}^{T}$ to preserve the original semantic information of each token to the fullest extent:

(9) $$\begin{array}{c} \{\begin{array}{c} R_{td}^{t}=D_{e}^{t}m_{t}^{T},\\ R_{tp}^{t}=P_{e}^{t}m_{t}^{T}. \end{array} \end{array}$$

Subsequently, we utilize bidirectional RNNs to fuse the vectors within the set to obtain overall vector representation. Then, we sum the diagnosis and procedure representation together to obtain drug-matching score from the token-level view, as follows:

(10) $$r_{t}^{t}={\text{RNN}}_{td}(R_{td}^{t})+{\text{RNN}}_{tp}(R_{tp}^{t}).$$

#### 2.2.3 Drug representation

Previous models typically recommend drugs based on learning patient representations, and the accuracy of patient representations may directly affect the accuracy of drug recommendations. Meanwhile, they primarily involve implicitly or coarsely calculating the similarity between a patient’s current and historical states, and then enhancing the current drug score vector by overall replicating the patient’s historical DR vector based on this similarity. These approaches fail to make selection decisions at the individual drug level, as the weights assigned to historical drugs suitable or unsuitable for the patient’s current state are bound together. Moreover, in clinical practice, some patients with chronic diseases may have specific medication treatments that accompany them for a lifetime. Based on Fig. 1, in both datasets, most of the current visit drugs have appeared in previous visits. Previous models do not screen useful drugs from the perspective of historical drugs. Based on the above motivation, we designed the DR module, which consists of gating drug retrieval and aggregation module.

**Gating drug retrieval (GDR):** We subtlety utilize historical information directly from the perspective of drug level. Concretely, MGRN develops a gating drug retrieval module to capture critical drug information from multiple visits, which can assist in recommending more reasonable drug combinations for the current visit. First, we concatenate the current visit and each historical visit to form a current history visit pair (CHVP). Then, we introduce a gating unit based on CHVP to store valuable gating memory representations. Subsequently, the gating memory vector appropriately assigns weights to historical drug labels resulting in a historical drug score vector.

To effectively model current and historical drug information, we concatenate the current DR vector with all historical DR vectors to generate a set $H^{t}$, composed of CHVP vectors:

(11) $$H^{t}=\overset{t-1}{\underset{i=1}{\left|\right|}}(C^{t}||C^{i}).$$

Subsequently, we introduce a gating unit based on CHVP set $H^{t}$ to store valuable gating DRs, obtaining historical medication score weight $G^{t}$ based on the current visit, where MLP denotes a multi layer perceptron and $G^{t}\in R^{(t-1)\times N_{m}}$. The calculation formula is as follows:

(12) $$G^{t}=\text{MLP}(H^{t}).$$

Then, we perform dot product operation between historical drug weights and historical drug sets to obtain the score of recommended drugs $r^{t}$, We represent the Gating Drug Retrieval as simply as the operator GDR(·), as follows:

(13) $$r^{t}=\sum_{i=1}^{t-1}G_{i}^{t}⊙M^{i},$$

where $M^{i}$ represents the set of drugs for the *i*-th visit.

**Aggregation module:** We utilize GDR(·) and input visit-level and sequence-level patient representations into GDR(·), respectively, to generate historical drug score vectors in vis- and sequence-visit views:

(14) $$r_{h}^{t}={\text{GDR}}_{v}(C_{v})+{\text{GDR}}_{s}(C_{s}).$$

Finally, we sum the drug-matching score vectors in the above three perspectives as the global perspective drug-match score vector:

(15) $$r_{g}^{t}=r_{v}^{t}+r_{s}^{t}+r_{t}^{t}.$$

In the aggregation module, we fuse and perform logistic regression on the global drug-matching score vector and historical drug-matching score vector to get the usage probability of each drug:

(16) $${\hat{o}}^{t}=\sigma (r_{g}^{t}+r_{h}^{t}).$$

Then, we select the drug based on the threshold ϕ and obtain the drug recommendation vector ${\hat{M}}^{t}$ for visit *t*.

(17) $$\begin{array}{c} {\hat{M}}_{i}^{t}=\{\begin{array}{c} 1({\hat{o}}_{i}^{t}\leq \phi ),\\ 0({\hat{o}}_{i}^{t}\leq \phi ), \end{array} \end{array}$$

where ϕ is a threshold to control whether a drug appears in the final drug prediction set.

#### 2.2.4 Loss function

Since MGRN is based on a multi-label binary classification task, we choose the binary cross-entropy loss $L_{\mathrm{bce}}$ and multi-label hinge loss (Ji and Ye 2009) $L_{\mathrm{mlh}}$ as the multi-label loss functions. Simultaneously, we utilize adaptive DDI loss $L_{\mathrm{addi}}$ to reduce the probability of adverse reactions between recommended drugs. Then, we combine the above three loss functions, and the joint loss can be expressed as follows:

(18) $$L=L_{\mathrm{bce}}+\alpha L_{\mathrm{mlh}}+\beta L_{\mathrm{addi}},$$

where α and β are the hyperparameters. Please refer to the Supplementary Materials for the detailed formula calculation.

#### 2.2.5 Algorithm

Our training algorithm is summarized in Algorithm S1.

## 3 Results and discussion

### 3.1 Datasets and evaluation indicators

The medical information mart for intensive care (MIMIC) dataset includes patients’ clinical data, such as laboratory tests, drug treatments, diagnostic codes, etc. It is widely used to develop and validate various medical algorithms and models, including predictive models and data mining. We conduct experiments based on two datasets, MIMIC-III (Johnson *et al*. 2016) and MIMIC-IV (Johnson *et al*. 2020). The specific data statistics are shown in the Supplementary Table S2.

Following the previous drug recommendation work (Yang *et al*. 2021a, 2021b), we utilize DDI rate, Jaccard similarity score (Jaccard), average *F*1 (*F*1), and precision recall AUC (PRAUC) as our evaluation indicators to assess the accuracy and effectiveness of our proposed model.

### 3.2 Baselines

To evaluate our MGRN, we select several popular drug recommendation models for comparison: ECC (Read *et al*. 2011), RETAIN (Choi *et al*. 2016), LEAP (Zhang *et al*. 2017), GAMENet (Shang *et al*. 2019), SafeDrug (Yang *et al*. 2021b), COGNet (Wu *et al*. 2022), DRMP (Ren *et al*. 2022), FFBDNet (Wang *et al*. 2022), DGCL (Li *et al*. 2023c), Carmen (Chen *et al*. 2023) and LEADER (Liu *et al*. 2024). Please refer to the Supplementary Materials for a detailed baseline introduction and implementation details.

### 3.3 Result analysis

#### 3.3.1 Overall comparison

Table 1 presents the results of all methods based on MIMIC-III and MIMIC-IV datasets, respectively. We conduct five rounds of testing for our models and present the average and standard deviation of their indicator scores. Our proposed MGRN model surpasses all comparison methods regarding higher Jaccard, PRAUC, and *F*1 scores.

**Table 1.** Performance comparison on the MIMIC-III/MIMIC-IV.

Methods	Jaccard	PRAUC	*F*1-score	DDI rate	AvgDrugs
ECC (Read *et al*. 2011)	0.4996/0.4510	0.7290/0.6844	0.6569/0.6007	0.0846/0.0762	18.0722/8.9866
RETAIN (Choi *et al*. 2016)	0.4887/0.4239	0.7556/0.6798	0.6481/0.5791	0.0835/0.0939	20.4051/10.8602
LEAP (Zhang *et al*. 2017)	0.4521/0.4287	0.6549/0.5506	0.6138/0.5820	0.0731/0.0592	18.7138/11.5198
GAMENet (Shang *et al*. 2019)	0.5067/0.4507	0.7631/0.7174	0.6626/0.6043	0.0864/0.0890	27.2147/18.4426
SafeDrug (Yang *et al*. 2021b)	0.5213/0.4651	0.7647/0.7118	0.6768/0.6117	0.0589/0.0740	19.9178/14.4705
COGNet (Wu *et al*. 2022)	0.5336/0.4884	0.7739/0.7087	0.6869/0.6367	0.0852/0.0894	28.0900/19.7235
DRMP (Ren *et al*. 2022)	0.5312/0.4913	0.7757/0.7338	0.6854/0.6435	0.0865/0.0872	22.7300/15.0512
FFBDNet (Wang *et al*. 2022)	0.5292/0.4970	0.7777/0.7435	0.6833/0.6454	0.0717/0.0838	19.6900/11.2906
DGCL (Li *et al*. 2023c)	0.5342/0.5000	0.7824/0.7506	0.6874/0.6550	**0.0587**/0.0735	28.6253/16.6284
Carmen (Chen *et al*. 2023)	0.5323/0.5049	0.7736/0.7513	0.6865/0.6615	–	–
LEADER (Liu *et al*. 2024)	0.5391/0.4779	0.7816/0.7120	0.6921/0.6296	–	–
MGRN	**0.5527/0.5261**	**0.7995/0.7749**	**0.7042/0.6796**	0.0649/0.0665	23.4712/21.0124

Numbers in bold and underlined indicate the previous and current best performance, respectively.

In detail, ECC and LEAP exhibit poor performance due to their reliance on instance-based modeling, focusing solely on the current visit. RETAIN, GAMENet, SafeDrug, DRMP, DGCL, and Carmen demonstrate relatively improved performance by incorporating longitudinal patient information in various manners. RETAIN and DMNC encode the patients’ historical data solely, while GAMENet introduces additional graph information, and SafeDrug integrates drug molecule structures, resulting in enhanced performance. Similar to SafeDrug, FFBDNet combines multiple medical knowledge to enhance the recommended effect. Carmen developed a context-sensitive graph neural network that integrates contextual data extracted from EHRs into molecular graphs. SafeDrug leverages additional drug molecule information to decrease the DDI rate. However, considering the average DDI rate of 0.08379 in the MIMIC-III dataset, our MGRN decreased by 1.89% compared to the dataset’s average DDI, indicating that our model can better imitate doctors in formulating reasonable prescriptions. On the MIMIC-III and MIMIC-IV datasets, compared to the latest application of the large language model LEADER, we outperform *F*1, PRAUC, and Jaccard indicators by *F*1, PRAUC, and Jaccard by 1.21%, 1.79%, 2.36%, and 5.0%, 6.29%, and 4.82%, respectively.

#### 3.3.2 Ablation experiments

To demonstrate the efficacy of each module of MGRN, we design the following ablation experiments: w/**O** vst: We remove the visit-level view. w/**O** seq: We remove the sequence-level view. w/**O** tkn: We remove the token-level view. **W**/o vst: We only use the visit-level view and remove the other two views. **W**/o seq: We only use the sequence-level view and remove the other two views. **W**/o tkn: We only use the token-level view and remove the other two views. w/**O** GDR: We remove the gating drug retrieval module and discard history drug information.

We design multiple MGRN variant models and conduct experiments on the MIMIC-III and MIMIC-IV datasets, and the results are shown in Table 2. The experimental results indicate that when only removing or using a particular view for patient–drug interaction, drug recommendation performance decreases. Especially when removing visit-level views, the performance indicators *F*1, PRAUC, and Jaccard showed the most significant decrease, with values of 3.57%, 3.1%, and 3.11%, respectively. This indicates the importance of historical medical information in obtaining early patient–drug interaction. The results of MGRN w/**O** GDR indicate that the history drugs also contribute to the final recommended performance. Overall, the complete MGRN outperforms all ablation models, which means each module of our model is valuable.

**Table 2.** Ablation study for different components of MGRN on MIMIC-III and MIMIC-IV.

	MIMIC-III			MIMIC-IV		
Methods	*F*1-score	PRAUC	Jaccard	*F*1-score	PRAUC	Jaccard
w/**O** vst	0.6731 ± 0.0011	0.7685 ± 0.0013	0.5170 ± 0.0008	0.6421 ± 0.0009	0.7381 ± 0.0011	0.4854 ± 0.0008
w/**O** seq	0.6849 ± 0.0012	0.7859 ± 0.0014	0.5308 ± 0.0009	0.6561 ± 0.0009	0.7546 ± 0.0013	0.5012 ± 0.0009
w/**O** tkn	0.6820 ± 0.0013	0.7811 ± 0.0016	0.5269 ± 0.0010	0.6511 ± 0.0012	0.7474 ± 0.0014	0.4951 ± 0.0010
**W**/o vst	0.6880 ± 0.0010	0.7823 ± 0.0012	0.5358 ± 0.0008	0.6572 ± 0.0008	0.7536 ± 0.0010	0.5022 ± 0.0007
**W**/o seq	0.6895 ± 0.0010	0.7875 ± 0.0011	0.5335 ± 0.0008	0.6551 ± 0.0009	0.7541 ± 0.0010	0.5002 ± 0.0008
**W**/o tkn	0.6864 ± 0.0012	0.7843 ± 0.0013	0.5323 ± 0.0009	0.6561 ± 0.0010	0.7526 ± 0.0011	0.5005 ± 0.0009
w/**O** GDR	0.6778 ± 0.0013	0.7738 ± 0.0015	0.5221 ± 0.0011	0.6447 ± 0.0011	0.7416 ± 0.0013	0.4883 ± 0.0010
MGRN	**0.7042 ± 0.0018**	**0.7995 ± 0.0017**	**0.5527 ± 0.0016**	**0.6796 ± 0.0012**	**0.7749 ± 0.0013**	**0.5261 ± 0.0010**

Numbers in bold indicate the best performance.

#### 3.3.3 Effect of history visit number

To further analyze whether our MGRN can more effectively capture historical drug data, we explore how the number of patient visits influences the performance of various models. We focus on the first five visits per patient in the test set, as most MIMIC patients visit the hospital fewer than five times. For comparison, we analyze the three strongest baselines, GAMENet, Safedrug, and DGCL, which also integrate historical information.

**Figure 3.**
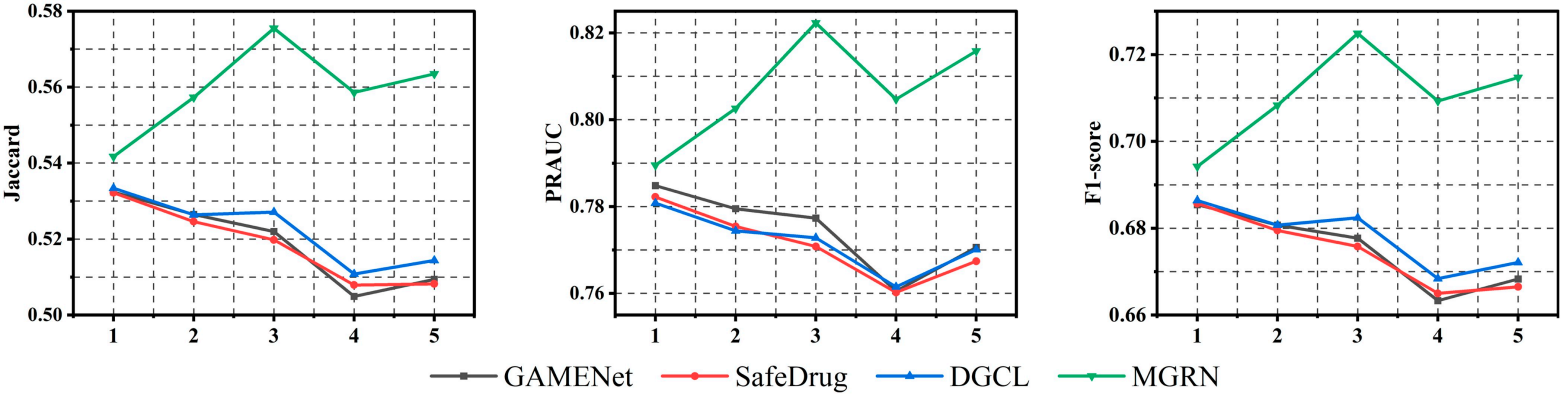
The effect of history visit number for different models based on the MIMIC-III dataset.

The results depicted in Fig. 3 and Supplementary Fig. S1, illustrate that MGRN exhibits comparatively superior performance as the number of visits increases based on both datasets. The remaining three models show a decreasing trend in all indicators on both datasets as the number of visits used accumulates. The possible reason may be that MGRN interacts with patients and drugs based on multiple views and replicates information from the perspective of historical drug sets. Both GAMENet and SafeDrug use RNN to model historical patient representations, which have a single perspective and weak temporal modeling ability of RNN. DGCL utilizes attention mechanisms to capture historical information, obtain a global representation of patients, and then map it to DRs, failing to model historical drugs directly.

#### 3.3.4 Robustness analysis

Medical data typically contains sensitive information and involves privacy protection, making it difficult to obtain electronic medical record data. To further demonstrate the MGRN’s ability to deal with a few EHRs, we design three sets of cases and give experimental results at multiple deletion rates. We then randomly delete 10%, 30%, and 50% of the training data on the MIMIC-III and MIMIC-IV datasets to conduct our proposed model MGRN’s robust.

**Figure 4.**
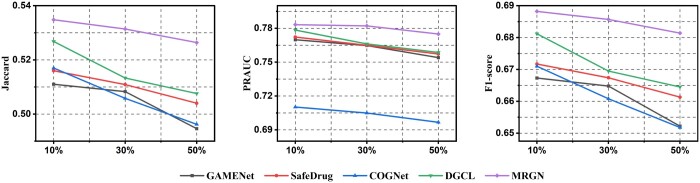
Robust analysis based on the MIMIC-III dataset. The horizontal axis represents the deletion rate of the dataset, with “10%,” “30%,” and “50%” deleted from left to right.

We select four mainstream drug recommendation models for comparative experiments. In Fig. 4 and Supplementary Fig. S2, as the deletion rate increases, all indicators of all models are attenuated to varying degrees. This indicates that the training data’s size affects the model’s overall training performance. Moreover, in the case of training with the same proportion of data, our model MGRN still has the highest performance. Especially under the same proportion of data loss changes, the performance degradation of MGRN is minimal. Under the same experimental setup, when the data undergoes varying degrees of disturbance, our model is more stable than other models on both datasets, indicating MGRN’s robustness.

In detail, especially on the MIMIC-III, as the number of training data decreases proportionally, COGENet exhibits the most significant performance fluctuations in Jacarard, PRAUC, and *F*1 scores. On the MIMIC-IV, the performance fluctuations of all models in Jacarard, PRAUC, and *F*1 scores are relatively small (except for the PRAUC indicator in COGNet). We suspect that it is because the data volume of MIMIC-IV far exceeds that of MIMIC-III, and when reducing the training data, the performance of each model does not experience a sharp decline. Overall, the above analysis indicates that our proposed model has competitive performance and robustness, especially with limited training data.

### 3.4 Case study

**Figure 5.**
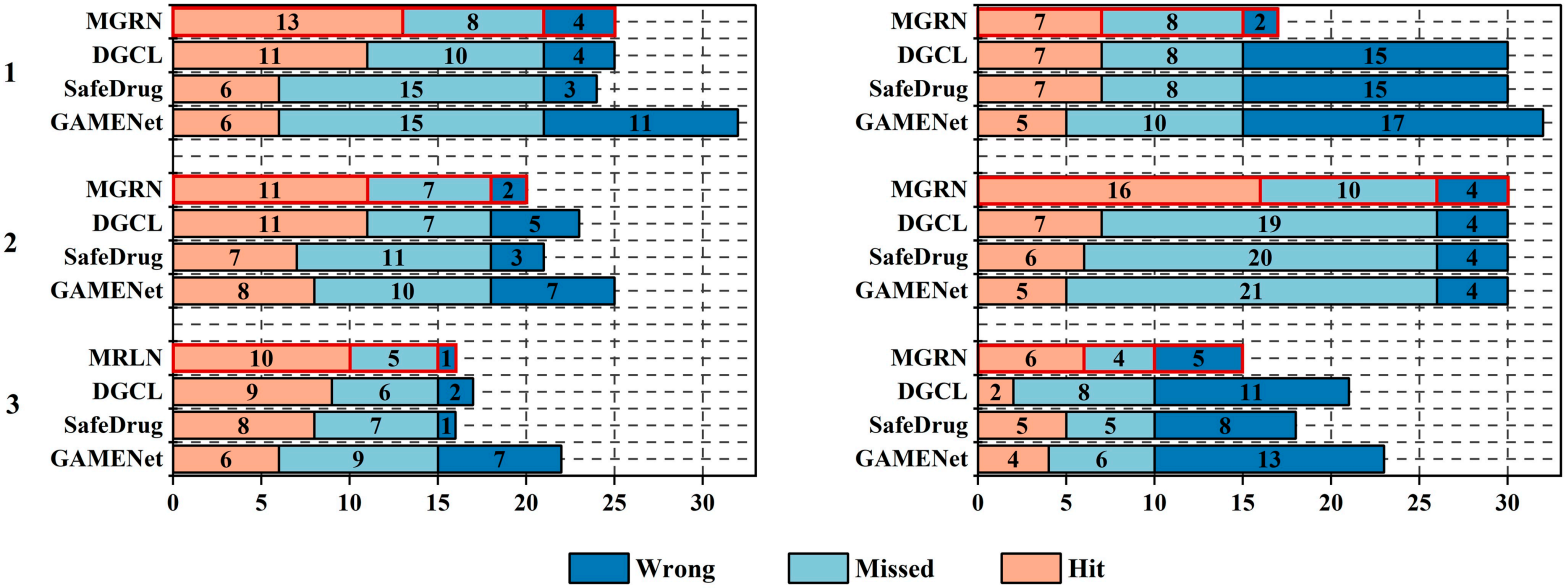
The overall performance on the cases of MGRN and other models on the MIMIC-III (left) and MIMIC-IV (right) datasets.

We design a case study to demonstrate our MGRN’s effectiveness. In detail, We choose a random visit of the three patients in the test set as our case study and leverage GAMENet, SafeDrug, COGNet, and DGCL to predict the drug combinations. The detailed diagnosis IDs, the drug ATC (Anatomical Therapeutic Chemical Classification System)-third code (in MIMIC-III or MIMIC-IV), and hit/missed/wrong drugs in each visit are provided in Supplementary Table S3. Moreover, we utilize Fig. 5 for a concise display. In Fig. 5 and Supplementary Table S3, the “missed” indicates the drugs in the ground truth but are not predicted, while the “wrong” refers to the drugs predicted by the models but are not in the ground truth, and the drugs under the “hit” are the ground truth label that the models predict.

Overall, MGRN shows a significant advantage in each patient’s case. Specifically, GAMENet exhibits higher overall unseen and wrong rates compared to other models because it encodes patients only in the visit view. SafeDrug is built upon GAMENet’s patient encoding approach, utilizes drug molecular structures to encode drugs in both local and global views, and shows slightly better performance than GAMENet. DGCL employs graph contrastive learning to alleviate contradictory relationships in the DDI graph and the EHR graph, thus achieving superior performance compared to the former two. Then on the MIMIC-IV dataset, MGRN demonstrates more pronounced advantages than the other models experimented on and improved based on the MIMIC-III dataset. We speculate that because the MIMIC-IV dataset has a larger label space compared to the MIMIC-III dataset, MGRN’s multi-view encoding mechanism can learn from different perspectives and accommodate feature related to drug labels more effectively.

## 4 Conclusion

We propose a MGRN for robust drug recommendation. We design visit-, sequence-, and token-level views to provide different perspectives on the interaction between patients and drugs, obtaining a more comprehensive representation of drugs. Moreover, we develop a gating drug retrieval module to capture critical drug information from multiple visits, which can assist in recommending more reasonable drug combinations for the current visit. We conduct extensive experiments on real-world MIMIC-III and MIMIC-IV datasets, and the results show that our proposed MGRN model achieves SOTA performance. Finally, we validate the robustness of our proposed model in the presence of training data perturbations.

### 4.1 Limitations and future work

Although the multi-view gated retrieval network we designed can significantly improve model performance, it cannot explicitly predict the process and lacks interpretability. In addition, with the rise of precision medicine, drug recommendations are no longer solely based on generic disease treatments but are more focused on individualized treatment plans. Introducing external knowledge, such as patient genomic information, pathological features, etc., will be a new research hotspot, which can make drug recommendations more accurate and improve treatment effectiveness.

## Supplementary Material

btae572_Supplementary_Data

## Data Availability

The datasets used in this paper are downloaded from https://mimic.physionet.org/.

## References

[btae572-B1] Chen Q , Li X, Geng K et al Context-aware safe medication recommendations with molecular graph and DDI graph embedding. AAAI 2023;37:7053–60.

[btae572-B2] Choi E , Bahadori MT, Sun J et al RETAIN: an interpretable predictive model for healthcare using reverse time attention mechanism. Adv Neural Inf Process Syst 2016;29:3504–12.

[btae572-B3] Hou X , Liu Z, Li X et al MKCL: medical knowledge with contrastive learning model for radiology report generation. J Biomed Inform 2023;146:104496. 37704104 10.1016/j.jbi.2023.104496

[btae572-B4] Ji S , Ye J. Linear dimensionality reduction for multi-label classification. In: *Twenty-First International Joint Conference on Artificial Intelligence*. Pasadena, California, USA: Elsevier, 2009, 1077–82.

[btae572-B5] Johnson A , Bulgarelli L, Pollard T et al MIMIC-IV. *PhysioNet*. 2020:49–55. https://physionet.org/content/mimiciv/1.0/. (23 August 2021, date last accessed).

[btae572-B6] Johnson AE , Pollard TJ, Shen L et al MIMIC-III, a freely accessible critical care database. Sci Data 2016;3:160035–9. 27219127 10.1038/sdata.2016.35 PMC4878278

[btae572-B7] Li X , Zhang Y, Hou X et al Multi-visit interactive recalibration network for drug recommendation with a triple graph encoder. In: *2023 IEEE International Conference on Bioinformatics and Biomedicine (BIBM)*. Istanbul, Turkiye: IEEE, 2023a, 2040–43.

[btae572-B8] Li X , Zhang Y, Li X et al NIDN: medical code assignment via note-code interaction denoising network. In: *Proceedings of 18th International Symposium on Bioinformatics Research and Applications (ISBRA)*. Haifa, Israel: Springer, 2023b, 62–74.

[btae572-B9] Li X , Zhang Y, Li X et al DGCL: distance-wise and graph contrastive learning for medication recommendation. J Biomed Inform2023c;139:104301. 36746345 10.1016/j.jbi.2023.104301

[btae572-B10] Li X , Liang S, Hou Y et al StratMed: relevance stratification between biomedical entities for sparsity on medication recommendation. Knowl-Based Syst 2024;284:111239.

[btae572-B11] Liu Q , Wu X, Zhao X et al Large language model distilling medication recommendation model. arXiv, arXiv:2402.02803, 2024, preprint: not peer reviewed.

[btae572-B12] Read J , Pfahringer B, Holmes G et al Classifier chains for multi-label classification. Mach Learn 2011;85:333–59.

[btae572-B13] Ren Y , Shi Y, Zhang K et al A drug recommendation model based on message propagation and DDI gating mechanism. IEEE J Biomed Health Inform 2022;26:3478–85. 35196249 10.1109/JBHI.2022.3153342

[btae572-B14] Shang J , Xiao C, Ma T et al GAMENet: graph augmented memory networks for recommending medication combination. AAAI 2019;33:1126–33.

[btae572-B15] Sun H , Xie S, Li S et al Debiased, longitudinal and coordinated drug recommendation through multi-visit clinic records. Adv Neural Inf Process Syst 2022;35:27837–49.

[btae572-B16] Wang L , Zhang W, He X et al Personalized prescription for comorbidity. In: Database Systems for Advanced Applications. Gold Coast, QLD, Australia: Springer, 2018, 3–19.

[btae572-B17] Wang Y , Chen W, Pi D et al Self-supervised adversarial distribution regularization for medication recommendation. In: IJCAI. Montreal, Canada: Elsevier, 2021a, 3134–40.

[btae572-B18] Wang Y , Chen W, Pi D et al Multi-hop reading on memory neural network with selective coverage for medication recommendation. In: *Proceedings of the 30th ACM International Conference on Information & Knowledge Management*. Queensland, Australia: ACM, 2021b, 2020–29.

[btae572-B19] Wang Z , Liang Y, Liu Z. FFBDNet: feature fusion and bipartite decision networks for recommending medication combination. In: *Joint European Conference on Machine Learning and Knowledge Discovery in Databases*. Grenoble, France: Springer, 2022, 419–36.

[btae572-B20] Wu J , Dong Y, Gao Z et al Dual attention and patient similarity network for drug recommendation. Bioinformatics 2023;39:btad003. 36617159 10.1093/bioinformatics/btad003 PMC9857978

[btae572-B21] Wu R , Qiu Z, Jiang J et al Conditional generation net for medication recommendation. In: *Proceedings of the ACM Web Conference 2022*. Lyon, France: ACM, 2022, 935–45.

[btae572-B22] Yang C , Xiao C, Glass L et al Change matters: medication change prediction with recurrent residual networks. In: *Proceedings of the International Joint Conference on Artificial Intelligence*. Montreal, Canada: Elsevier, 2021a, 3728–34.

[btae572-B23] Yang C , Xiao C, Ma F et al SafeDrug: dual molecular graph encoders for safe drug recommendations. In: *Proceedings of the International Joint Conference on Artificial Intelligence*. Montreal, Canada: Elsevier, 2021b, 3735–41.

[btae572-B24] Zhang Y , Chen R, Tang J et al LEAP: learning to prescribe effective and safe treatment combinations for multimorbidity. In: *Proceedings of the 23rd ACM SIGKDD International Conference on Knowledge Discovery and Data Mining*. Halifax, NS, Canada: ACM, 2017, 1315–24.

