# Supplementary material for: MGRN: toward robust drug recommendation via multi-view gating retrieval network
Source: Bioinformatics. 2024 Sep 24;40(10):btae572. doi: 10.1093/bioinformatics/btae572 (PMC11470236; doi:10.1093/bioinformatics/btae572)
Supplement: btae572_Supplementary_Data [file btae572_supplementary_data.pdf]

Bioinformatics, vol(num), 2024, 1–5
doi: 10.1093/bioinformatics/xxxxxx
Article

MGRN: towards robust drug recommendation via multi-view gating retrieval network

Fanjun Meng [1,†], Xiaobo Li [2,†], Xiaodi Hou[1], Mingyu Lu[1], and Yijia Zhang [2,*]

[1] School of Artificial Intelligence, Dalian Maritime University, 116026, China.
[2] School of Information Science and Technology, Dalian Maritime University, 116026, China.

[*] Corresponding author: zhangyijia@dlmu.edu.cn
[†] Contributed equally to this manuscript

Supplementary materials

A RELATED WORKS

Existing strategies for medication recommendation can generally be divided into two primary categories: instance-based and longitudinal-based. Instance-based methods [Wang et al., 2018, Zhang et al., 2017] focus solely on information from the current visit. For example, LEAP [Zhang et al., 2017] treats medication recommendation as a sequential decision-making process, utilizing a recurrent decoder to capture label relationships during the current visit. However, these methods overlook the historical visit data of the patient. On the other hand, longitudinal-based approaches [Li et al., 2023a, Yang et al., 2023] integrate temporal visit records to extract crucial information that can enhance patient representation. For instance, DMNC [Le et al., 2018] leverages a memory-augmented network to capture asynchronous interactions and long-term dependencies across different views. Similarly, MICRON [Yang et al.] employs a recurrent residual network to account for medication changes across different visits. COGNet [Wu et al., 2022] introduces a hierarchical selection mechanism to integrate past visit information, enhancing the interpretability of recommended medications. Despite advancements in deep learning providing promising solutions for medication recommendations, these approaches often fail to establish a connection between medications used in previous visits and those recommended for the current visit.

Table S1: Notations used in MGRN

Notation	Description
N_d	The number of diagnose labels
N_p	The number of procedure labels
N_m	The number of drug labels
d	The embedding dimension
T	The number of visit
$D \in \{0,1\}^{N_d}$	The diagnose sequence
$P \in \{0,1\}^{N_p}$	The procedure sequence
$M \in \{0,1\}^{N_m}$	The drug sequence
$C_* \in \mathbb{R}^{2d}$	The patient representation
$r_* \in \mathbb{R}^{N_m}$	The drug-matching score
$A \in \mathbb{R}^{N_m \times N_m}$	The DDI adjacency matrix
$\hat{o} \in \mathbb{R}^{N_m}$	The drug probability vector
$\hat{O} \in \mathbb{R}^{T \times N_m}$	The drug probability vector
$M \in \mathbb{R}^{N_m}$	The ground truth of drug labels
$\hat{M} \in \mathbb{R}^{N_m}$	The predicted drug labels
σ	Sigmoid activation function
ϕ	The threshold for the outputs

B PROBLEM FORMULATION AND METHODS

Loss function

Binary cross-entropy loss.

The $\mathcal{L}_{bce}$ can be formulated as:

$$\mathcal{L}_{bce} = \sum_{t=1}^{T}\sum_{i=1}^{N_m}[M_i^t \log(\hat{o}_i^t) + (1 - M_i^t)\log(1 - \hat{o}_i^t)] \tag{S1}$$

Multi-label hinge loss.

The $\mathcal{L}_{mlh}$ is used to ensure there is sufficient margin

between positive and negative examples in the output:

$$\mathcal{L}_{mlh} = \sum_{t=1}^{T} \sum_{M_i^t=1, M_j^t=1} [\frac{max(0, 1-(\hat{o}_i^t - \hat{o}_j^t))}{|M^t|}] \tag{S2}$$

Adaptive DDI loss.

We set the DDI graph as an adjacency matrix A, where elements with a value of 1 represent the presence of DDI relationships between the drug corresponding to the horizontal index and the drug corresponding to the vertical index. Then we exclude drug pairs from the DDI matrix A that are also present in the current drug set M to allow the DDI loss $\mathcal{L}_{addi}$ to adaptively avoid conflicts with the EHR:

$$\mathcal{L}_{addi} = \sum_{i=1}^{T} \sum_{j=1}^{N_m} \sum_{i=1}^{N_m} \hat{o}^t \cdot [A_{i,j} - M_{i,j}^t \cdot A_{i,j}] \tag{S3}$$

Algorithm

Our training algorithm is summarized in Algorithm S1.

Algorithm S1 One training epoch of MGRN

Input: Training set: $\{Patient\}_{i=1}^{N}$; DDI matrix A.
Initialize the parameters in: $Embedding_d$, $Embedding_p$, M_v, M_s, M_t, RNN$_{vd}$, RNN$_{vp}$, RNN$_{sd}$, RNN$_{sp}$, RNN$_{td}$, RNN$_{tp}$, MG$_v$, MG$_s$, GDR$_v$, GDR$_s$
for *Patient* $i = 1$ *to* N **do**
  Initialize an empty set O_i for patient i to contain the outputs of each visit
  for *Visit* $t = 1$ *to* T **do**
    Project the diagnosis and procedure labels into the embedding space and obtain embedding representations D_e^t and P_e^t
    Generate patient representations C_v^t and C_s^t
    Calculate the historical drug score r_h^t in vis- and sequence- views
    Calculate the visit level perspective drug-matching score r_v^t
    Calculate the sequence level perspective drug-matching score r_s^t
    Generate the token level perspective drug-matching score r_t^t
    Calculate the drug usage probability vector $\hat{o}^t$
    Calculate the predicted drug labels $\hat{M}^t$ by ϕ
    Append $\hat{o}^t$ into the patient visit set O_i
  end
  Calculate the loss between O_i and M_i
  Optimize the loss
end

Table S2: Statistics of MIMIC-III and MIMIC-IV

Item	MIMIC-III	MIMIC-IV
#Patients	6,350	8,546
#Clinical events	15,032	19,592
#Diagnoses	1,958	1,892
#Procedures	1,430	4,798
#Medicines	131	131
Avg/max #of visits	2.37/29	2.29/28
Avg/max #of diagnoses	13.63/39	17.37/39
Avg/max #of procedures	4.53/32	4.02/34
Avg/max #of medicines	19.19/53	16.74/64

C EXPERIMENTS AND RESULTS

Baselines

- ECC [Read et al., 2011] designs a classifier chain approach to capture label dependency.
- RETAIN [Choi et al., 2016] designs reverse time attention model to enhance the model's interpretability.
- LEAP [Zhang et al., 2017] considers treatment recommendations as a sequential decision-making challenge that leverages a recurrent decoder to capture label dependencies and employs content-driven attention to model the intricate mapping between labels and individual instances.
- GAMENet [Shang et al., 2019] incorporates the knowledge graph of drug-drug interactions through a memory component realized as graph convolutional networks and represents the longitudinal patient records as the input queries for analysis.
- SafeDrug [Yang et al., 2021] utilizes the molecular structures of drugs and explicitly models drug-drug interactions (DDIs) to enhance the safety of drug recommendations.
- COGNet [Wu et al., 2022] designs a hierarchical selection mechanism to choose the reusable medications to copy from different perspectives.
- DRMP [Ren et al., 2022] utilizes a message propagation mechanism to improve the confidence of the recommended drugs.
- FFBDNet [Wang et al., 2022] designs a feature fusion and bipartite decision network to focus the fusion of heterogeneous knowledge.
- DGCL [Li et al., 2023b] formulates a distance-based detection loss mechanism to assess the degree of simi-

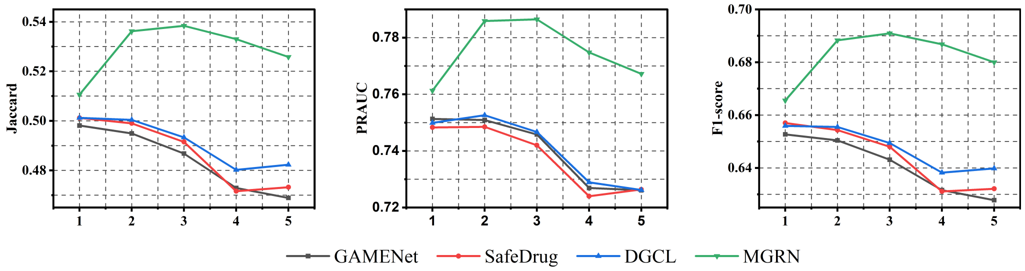

Figure S1: The effect of history visit number for different models based on the MIMIC-IV dataset.

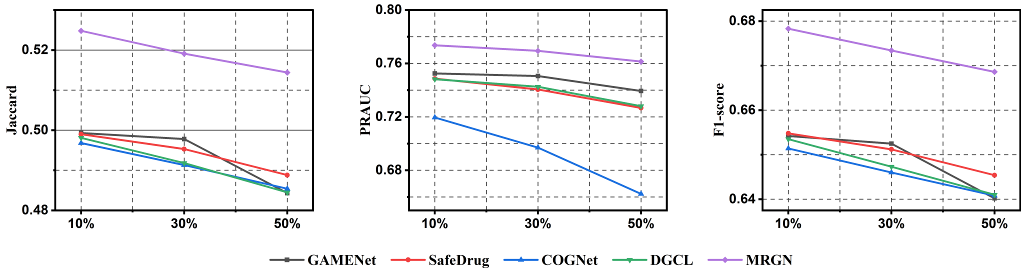

Figure S2: Robust analysis based on the MIMIC-IV dataset. The horizontal axis represents the deletion rate of the dataset, with "10%", "30%", and "50% " deleted from left to right.

larity between the present condition and prior visits and leverages graph contrastive learning to mitigate drug-drug interactions.

• Carmen [Chen et al., 2023] constructs a context-aware graph neural network that incorporates contextual information extracted from electronic health records (EHRs) into molecular graph structures, empowering it to generate more distinctive representations of drugs.

• LEADER [Liu et al., 2024] integrates a large language model to recommend drugs.

Implementation details

According to the existing work [Wu et al., 2022, Yang et al., 2021], we divide the dataset into training, validation, and test sets at a 4:1:1 ratio. Our model is implemented using Python 3.11 and PyTorch 1.19.2, and training and testing conduct on Intel Gen CPU and NVIDIA 3080Ti GPU. The optimal hyperparameters are selected for the model with the embedding dimension size set at 64. For the varying dataset sizes, we set the learning rate of 0.0001 for both datasets.

Table S3: Case study and detailed data of MRGN on MIMIC-III and MIMIC-IV

Dataset	Patient	Method	Hit	Missed	Wrong
MIMIC-III	1	GAMENet	N02B, A01A, A02B, A06A, B05C, A12C	A07A, A12B, N02A, B01A, N05C, N03A, N07B, D06A, D04A	A12A, C03C, N07A, N06A, A11C, N05A, M03A
		SafeDrug	N02B, A01A, A02B, A06A, B05C, A12C, A12B, B01A	A07A, N02A, N05C, N03A, N07B, D06A, D04A	C07A
		DGCL	N02B, A01A, A02B, A06A, B05C, A12C, A07A, A12B, N02A	N05C, N07B, D06A, D04A, B01A, N03A	A12A, C03C
		MGRN	N02B, A01A, A02B, A06A, B05C, A12C, A12B, N02A, B01A, N03A	A07A, N05C, N07B, D06A, D04A	N05B
	2	GAMENet	A02B, A06A, B05C, A12A, A12C, C07A, C03C, A12B	N02B, N01A, J01M, N05C, B02B, R01A, D04A, C03D, J01C, H01C	A01A, A07A, N07A, C02A, C09A, R06A, C05A
		SafeDrug	N02B, A02B, A06A, B05C, A12C, C07A, A12B	A12A, N01A, C03C, J01M, N05C, B02B, R01A, D04A, C03D, J01C, H01C	A01A, C01C, B01A
		DGCL	A02B, A06A, B05C, A12A, A12C, N01A, C07A, C03C, A12B, J01M, N05C	N02B, R01A, D04A, B02B, C03D, J01C, H01C	A01A, C01C, A07A, R03A, B03B
		MGRN	N02B, A02B, A06A, B05C, A12A, A12C, N01A, C03C, A12B, N05C, H01C	C07A, J01M, B02B, R01A, D04A, C03D, J01C	A01A, R03A
	3	GAMENet	N02B, A01A, A02B, A06A, A12C, A07A	N02A, B01A, A03B, C10A, J01D, A04A, A07E, R03A, D07A, N05B, A03F, R01A, C05A, J01C, J01G	B05C, C03C, A12B, N07A, N06A, A02A, A11C, B02B, N03A, B03B, G04B
		SafeDrug	N02B, A01A, A02B, A06A, A12C, B01A	A07A, N02A, A03B, C10A, J01D, A04A, A07E, R03A, D07A, N05B, A03F, R01A, C05A, J01C, J01G	B05C, C07A, A12B
		DGCL	N02B, A01A, A02B, A06A, A12C, A07A, B01A, C10A, J01D, R03A, R01A	N02A, A03B, A04A, A07E, D07A, N05B, A03F, C05A, J01C, J01G	B05C, C07A, C03C, A12B
		MGRN	N02B, A01A, A02B, A06A, A12C, A07A, N02A, B01A, C10A, J01D, A04A, R03A, R01A	A03B, A07E, D07A, N05B, A03F, C05A, J01C, J01G	C07A, C03C, A12B, B03B
MIMIC-IV	1	GAMENet	A06A, N02B, A12C, B01A, B05C, A12A, A12B	J01D, J01M, A02A, C03A, D07A	N02A, A01A, C07A
		SafeDrug	A06A, N02B, A12C, B01A, B05C, J01D, A12A, A12B, A02A	J01M, C03A, D07A	N03A, N02A, A07A, A01A, A02B, R03A, A04A, N05B
		DGCL	A06A, N02B, A12C, B01A, B05C, J01D	J01M, C03A, D07A, A12A, A12B	N02A, A01A, A07A
		MGRN	A06A, N02B, A12C, B01A, B05C, J01D, A12A, A12B, A02A	J01M, C03A, D07A	A01A, A02B
	2	GAMENet	A06A, N02B, B01A, C01B, B05C, A01A, C07A, C03C, A12B, C01C	N03A, N02A, A12C, C10A, R01A, N06A, N05C, M04A, C03D, D07A, C01D, C09C	A02B, R03A, N05B
		SafeDrug	A06A, N02B, A12C, B01A, C01B, B05C, A01A, C07A, C03C, A12B	N03A, N02A, C10A, R01A, N06A, N05C, C01C, M04A, C03D, D07A, C01D, C09C	A02B, N05B, A02A, C09A
		DGCL	A06A, N02B, B01A, C01B, B05C, A01A, C10A, C07A, C03C, A12B, C01C, C03D	N03A, N02A, A12C, R01A, N06A, N05C, M04A, D07A, C01D, C09C	A02B, R03A, C09A
		MGRN	A06A, N02B, N02A, A12C, B01A, C01B, B05C, A01A, C10A, R01A, N06A, C07A, C03C, A12B, M04A, C03D, D07A, C01D, C09C	N03A, N05C, C01C	A02B, A02A
	3	GAMENet	A06A, N03A, N02B, A12C, C02D, B05C, C07A	C01B, A12B, A02A, C08C	N02A, B01A, A01A, A02B, C10A
		SafeDrug	A06A, N03A, N02B, A12C, C02D, B05C, C07A, A12B	C01B, A02A, C08C	B01A, A01A, J01D, A02B, N05A
		DGCL	A06A, N03A, N02B, A12C, C02D, B05C, C07A, A12B, C08C	C01B, A02A	B01A, A01A, J01D, A02B
		MGRN	A06A, N03A, N02B, A12C, C02D, B05C, C07A, A12B, A02A, C08C	C01B	N02A, B01A, J01D, A02B

References

1. Qianyu Chen, Xin Li, Kunnan Geng, and Mingzhong Wang. Context-aware safe medication recommendations with molecular graph and ddi graph embedding. In *Proceedings of the AAAI Conference on Artificial Intelligence*, volume 37, pages 7053–7060, 2023.
2. Edward Choi, Mohammad Taha Bahadori, Jimeng Sun, Joshua Kulas, Andy Schuetz, and Walter Stewart. Retain: An interpretable predictive model for healthcare using reverse time attention mechanism. *Advances in neural information processing systems*, 29, 2016.
3. Hung Le, Truyen Tran, and Svetha Venkatesh. Dual memory neural computer for asynchronous two-view sequential learning. In *Proceedings of the 24th ACM SIGKDD international conference on knowledge discovery & data mining*, pages 1637–1645, 2018.
4. Xiaobo Li, Yijia Zhang, Xiaodi Hou, Fanjun Meng, and Hongfei Lin. Multi-visit interactive recalibration network for drug recommendation with a triple graph encoder. In *2023 IEEE International Conference on Bioinformatics and Biomedicine (BIBM)*, pages 2040–2043. IEEE, 2023a.
5. Xingwang Li, Yijia Zhang, Xiaobo Li, Hao Wei, and Mingyu Lu. Dgcl: Distance-wise and graph contrastive learning for medication recommendation. *Journal of Biomedical Informatics*, 139:104301, 2023b.
6. Qidong Liu, Xian Wu, Xiangyu Zhao, Yuanshao Zhu, Zijian Zhang, Feng Tian, and Yefeng Zheng. Large language model distilling medication recommendation model. *arXiv preprint arXiv:2402.02803*, 2024.
7. Jesse Read, Bernhard Pfahringer, Geoff Holmes, and Eibe Frank. Classifier chains for multi-label classification. *Machine learning*, 85:333–359, 2011.
8. Yongjian Ren, Yuliang Shi, Kun Zhang, Xinjun Wang, Zhiyong Chen, and Hui Li. A drug recommendation model based on message propagation and ddi gating mechanism. *IEEE Journal of Biomedical and Health Informatics*, 26(7):3478–3485, 2022.
9. Junyuan Shang, Cao Xiao, Tengfei Ma, Hongyan Li, and Jimeng Sun. Gamenet: Graph augmented memory networks for recommending medication combination. In *proceedings of the AAAI Conference on Artificial Intelligence*, volume 33, pages 1126–1133, 2019.
10. Lu Wang, Wei Zhang, Xiaofeng He, and Hongyuan Zha. Personalized prescription for comorbidity. In *Database Systems for Advanced Applications*, pages 3–19. Springer, 2018.
11. Zisen Wang, Ying Liang, and Zhengjun Liu. Ffbdnet: Feature fusion and bipartite decision networks for recommending medication combination. In *Joint European Conference on Machine Learning and Knowledge Discovery in Databases*, pages 419–436. Springer, 2022.
12. Rui Wu, Zhaopeng Qiu, Jiacheng Jiang, Guilin Qi, and Xian Wu. Conditional generation net for medication recommendation. In *Proceedings of the ACM Web Conference 2022*, pages 935–945, 2022.
13. Chaoqi Yang, Cao Xiao, Lucas Glass, and Jimeng Sun. Change matters: Medication change prediction with recurrent residual networks. In *In Proceedings of the International Joint Conference on Artifcial Intelligence*, pages 3728–3734.
14. Chaoqi Yang, Cao Xiao, Fenglong Ma, Lucas Glass, and Jimeng Sun. Safedrug: Dual molecular graph encoders for safe drug recommendations. In *Proceedings of the International Joint Conference on Artifcial Intelligence*, pages 3735–3741, 2021.
15. Nianzu Yang, Kaipeng Zeng, Qitian Wu, and Junchi Yan. Molerec: Combinatorial drug recommendation with substructure-aware molecular representation learning. In *Proceedings of the ACM Web Conference 2023*, pages 4075–4085, 2023.
16. Yutao Zhang, Robert Chen, Jie Tang, Walter F Stewart, and Jimeng Sun. Leap: learning to prescribe effective and safe treatment combinations for multimorbidity. In *proceedings of the 23rd ACM SIGKDD international conference on knowledge Discovery and data Mining*, pages 1315–1324, 2017.
